# The Quadruplex Real‐Time PCR Assay for Simultaneous Detection of GETV, PRRSV, JEV, and PCV2 in Swine

**DOI:** 10.1155/tbed/7291266

**Published:** 2026-06-11

**Authors:** Haojie Wang, Lihong Xue, Bowen Shan, Shuyan Wu, Tongqin An, Changqing Yu, Changyou Xia, He Zhang

**Affiliations:** ^1^ State Key Laboratory of Animal Disease Control and Prevention, Harbin Veterinary Research Institute, Chinese Academy of Agricultural Sciences, Harbin, China, caas.cn; ^2^ School of Advanced Agricultural Sciences, Yibin Vocational and Technical College, Yibin, China

**Keywords:** getah virus, japanese encephalitis virus, porcine circovirus type 2, porcine reproductive and respiratory syndrome virus, real-time PCR

## Abstract

This study aimed to establish a quadruplex real‐time PCR assay for the simultaneous detection of Getah virus (GETV), Porcine reproductive and respiratory syndrome virus (PRRSV), Japanese encephalitis virus (JEV), and Porcine circovirus type 2 (PCV2). Specific primers and probes were designed based on comparative analysis of conserved gene sequences from each pathogen. The optimal reaction system and conditions were determined through systematic optimization, standard curves were established, and the assay’s specificity, sensitivity, repeatability, and clinical application performance were evaluated. The standard curves all exhibited good linear relationships, with *R* ^2^ values of 0.9993, 0.9972, 0.9976, and 0.9995, respectively. The amplification efficiencies were 101.286%, 109.863%, 98.626%, and 122.211%, respectively. The limits of detection (at 95% confidence) for GETV, PRRSV, JEV, and PCV2 were 7.454, 7.111, 24.876, and 7.7 copies, respectively. The results demonstrated no cross‐reactivity with 12 other common swine pathogens, including pseudorabies virus (PRV) and classical swine fever virus (CSFV). Both intra‐assay and inter‐assay coefficients of variation (CV) were below 1.4%. Testing of 151 clinical samples using this assay revealed positive rates of 18.54%, 14.57%, 3.31%, and 10.60% for GETV, PRRSV, JEV, and PCV2, respectively, with 100% concordance compared to established standard methods. The developed quadruplex real‐time PCR assay offers advantages of high throughput, sensitivity, specificity, and stability, providing a technical foundation for rapid differential diagnosis and epidemiological surveillance of GETV, PRRSV, JEV, and PCV2 in swine populations.

## 1. Introduction

Getah virus (GETV), belonging to the genus Alphavirus of the family Togaviridae, is an important zoonotic virus that is mainly transmitted by arthropod vectors and is an important pathogenic microorganism that cannot be ignored in terms of its threat to public health and the development of animal husbandry [1]. GETV infection can cause illness in equine animals and also affect pigs of different age groups [2, 3]. Newborn and suckling piglets infected with GETV may exhibit symptoms such as high fever, diarrhea, and paralysis and even die. Pregnant sows infected with GETV may experience miscarriage and stillbirth and can also infect piglets through vertical transmission, seriously affecting the reproductive capacity of breeding pigs [2]. In recent years, it has broken out and become prevalent in places such as China, posing a potential major threat to the pig‐farming industry [2, 4, 5]. Timely and accurate clinical differential diagnosis can effectively prevent and control the spread of GETV.

The pathogenic microorganisms that can cause reproductive disorders in pigs, apart from GETV, include the porcine reproductive and respiratory syndrome virus (PRRSV) [6, 7]. PRRSV is a single‐stranded positive‐sense RNA virus with a capsid. After pigs of all ages are infected with PRRSV, they generally exhibit symptoms such as fever, listlessness, decreased appetite, breathing difficulties, and cyanosis of the body surface, skin, and ears. In addition to the above symptoms, clinical manifestations of pregnant sows infected with PRRSV also include premature birth, abortion, stillbirth, and even death [7]. Therefore, when conducting the detection of GTEV, the first step is to carry out a differential diagnosis to rule out PRRSV infection. Currently, PRRSV is showing a global epidemic trend, posing a huge challenge to the prevention and control of clinical diseases and continuously affecting the healthy development of the pig‐farming industry [8]. Apart from PRRSV, another virus that cannot be overlooked in clinical diagnosis and which can cause reproductive disorders is porcine Circovirus 2 (PCV2), which belongs to the Circovirus family and the Circovirus genus [9]. The symptoms of PCV2 are diverse and complex, mainly including reproductive disorders in sows, multisystem failure syndrome in weaned piglets, and respiratory diseases in pigs [10]. The clinical symptoms caused by PCV2 can become more complicated due to coinfection with other pathogenic microorganisms such as PRRSV [11]. It is also often an important virus that is easily overlooked in the clinical diagnosis of GETV. Moreover, infection with Japanese encephalitis virus (JEV) can lead to reproductive disorders such as abortion and stillbirth in pregnant sows [12]. JEV belongs to the Flaviviridae family and the Flavivirus genus, and infected piglets often show neurological symptoms. JEV and GETV are both zoonotic viruses transmitted by insects [13]. Therefore, during the peak season of mosquito activity, there is often a possibility of cotransmission of these two viruses, making them important misleading pathogens for the differential diagnosis of GETV.

In conclusion, GETV, PRRSV, PCV2, and JEV all cause reproductive disorders in pregnant sows in clinical settings. When diagnosing clinical cases of reproductive disorders such as abortion and stillbirth, it is necessary to consider the possibility of these four viruses simultaneously. Research reports have also found that cases of mixed infection of these four viruses occur from time to time [14–16]. Therefore, it is difficult to conduct timely and accurate diagnoses solely based on clinical symptoms and single‐pathogen detection methods. Therefore, it is urgent to develop a diagnostic tool that can quickly, accurately, and simultaneously detect these four pathogens. The current methods used for rapid clinical diagnosis include antigen detection and antibody detection [17, 18]. The fluorescence quantitative PCR method is currently recognized as the most reliable means for rapid, accurate, and economical antigen detection [15]. This study aims to develop and optimize a multiplex real‐time PCR method capable of simultaneously detecting GETV, PRRSV, PCV2, and JEV. The establishment of this method is intended to provide an integrated technical solution for the rapid clinical diagnosis of these four key pathogens, the investigation of mixed infections, epidemiological monitoring, and the evaluation of farm purification. It holds significant practical importance for ensuring the healthy and stable development of the pig‐farming industry.

## 2. Materials and Methods

### 2.1. Nucleic Acids of Viruses and Bacteria

Specific control nucleic acids, including GETV, PRRSV, JEV, PCV2, *Streptococcus suis* (*S. suis*), *Glaesserella parasuis* (*G. parasuis*), porcine transmissible gastroenteritis virus (TGEV), porcine epidemic diarrhea virus (PEDV), porcine rotavirus (RVA), *Escherichia coli* (*E. coli*), *Salmonella*, pseudorabies virus (PRV), porcine sapelovirus (PSV), porcine parvovirus (PPV), and classical swine fever virus (CSFV), were maintained in our laboratory.

### 2.2. Design of Primers and Probes

Representative full‐genome sequences of GETV, PRRSV, JEV, and PCV2 strains circulating in different regions worldwide in recent years were downloaded from the NCBI GenBank database. These sequences were aligned and analyzed using MegAlign software to select highly conserved and specific nucleotide regions as targets. Primers and probes (Table 1) were designed using Primer Express 3.0.1 software. The probes for GETV, PRRSV, JEV, and PCV2 were labeled at the 5′ end with FAM, ROX, Cy5, and JED fluorophores, respectively. All probes were labeled with a minor groove binder (MGB) quencher at the 3′ end. All primers and probes were synthesized by Sangon Biotech (Shanghai) Co., Ltd., with a working concentration of 10 μM.

**Table 1:** Sequences of primers and probes.

Pathogen	Primers and probes (sequences 5′‐3′)	Product size
GETV	F: TGTTGCCTGTGGGCGTTTA	120 bp
	R: TGACCACAGGCTCGGTATCA	
	Probe: FAM‐CCCACACCATGTATAAG‐MGB	
PRRSV	F: TTAAACTGYTAGCCGYCAG	76 bp
	R: ATYTTTACCGCYGTYTCAGT	
	Probe: ROX‐YYTGACCCGCTGTGGY‐MGB	
JEV	F: ACCAGGGAAACCTGCAGTAAAC	95 bp
	R: CCCGTGGGTAGTCCAAGCT	
	Probe: Cy5‐CCAGACAAAACCGGGAGT‐MGB	
PCV2	F: TGACCTCTCTACTGCTGTGAGTACCT	100 bp
	R: CAGCCCGCGGAAATTTCT	
	Probe: JED‐TCTGGTGACCGTTGCAG‐MGB	

### 2.3. Preparation of Recombinant Plasmid Standards

The conserved and specific target sequences of GETV, PRRSV, JEV, and PCV2 were downloaded and synthesized and then constructed into the pMD18T vector by Sangon Biotech (Shanghai) Co., Ltd. These four recombinant positive plasmid standards were designated as pMD18T‐GETV, pMD18T‐PRRSV, pMD18T‐JEV, and pMD18T‐PCV2, respectively. The concentration of each recombinant plasmid standard was measured using a NanoDrop Microplate Spectrophotometer (Thermo Fisher Scientific, USA) and converted to copy number based on the appropriate formula [19].

### 2.4. Optimization of Quadruplex Real‐Time PCR Conditions

The annealing temperature (58–62°C), primer concentration (0.1–0.5 µM), probe concentration (0.1–0.5 µM), and cycle number (35, 40, 45, and 50) for the quadruplex real‐time PCR were optimized using matrix and single‐factor‐at‐a‐time approaches. The optimal reaction conditions were determined based on the criteria of the smallest Ct value and the strongest fluorescence signal.

### 2.5. Establishment of Standard Curves

First, the standard recombinant plasmids of pMD18T‐GETV, pMD18T‐PRRSV, pMD18T‐JEV, and pMD18T‐PCV2 were mixed to ensure that the final concentration of each recombinant plasmid standard is 1 × 10^9^ copies/µL. Then, tenfold serial dilutions were performed. A concentration range from 1 × 10^9^ to 1 × 10^2^ copies/µL for each was selected as the template for amplification using the optimized quadruplex real‐time PCR assay. After the reaction was completed, the copy number of the positive plasmid standard was taken as the abscissa and the Ct value as the ordinate. The standard curve was exported from QuantStudio 5 software (Thermo Fisher Scientific, USA), and *R* ^2^ and amplification efficiency were calculated.

### 2.6. Sensitivity Test

The recombinant plasmid standards (pMD18T‐GETV, pMD18T‐PRRSV, pMD18T‐JEV, and pMD18T‐PCV2) were serially diluted tenfold from 1 × 10^9^ copies/µL to 1 × 10^0^ copy/µL using nuclease‐free water, followed by testing with the established quadruplex real‐time PCR assay. Subsequently, twofold serial dilutions were prepared (with concentrations set at 50, 25, 12.5, 6.25, 3.125, and 1.5625 copies per reaction for GETV, PRRSV, and PCV2 and at 200, 100, 50, 25, 12.5, and 6.25 copies per reaction for JEV). These dilutions were then tested using the established quadruplex real‐time PCR assay, with each dilution tested in 40 replicates. Detection rates were calculated for each concentration, and probit regression analysis was performed using SPSS software to determine the limit of detection (LOD) of the method for each pathogen [7].

Plasmid copies/µL=6.02×1023×X × ng/µL×10−9/constructed plasmid lengthbp×660.

### 2.7. Specificity Test

The specificity of the established quadruplex real‐time PCR assay was evaluated by testing nucleic acids from nontarget pathogens: *S. suis*, *G. parasuis*, TGEV, PEDV, RVA, *E. coli*, *Salmonella*, PRV, PPV, and CSFV. GETV, PRRSV, JEV, and PCV2 were used as positive controls, while nuclease‐free water served as the negative control. This test assessed the potential cross‐reactivity of the assay with nontarget pathogens.

### 2.8. Repeatability Test

Recombinant plasmid standards at concentrations of 1 × 10^7^, 1 × 10^5^, and 1 × 10^3^ copies/µL were selected as templates. Intra‐assay and inter‐assay repeatability tests were performed using the optimized quadruplex real‐time PCR method. The mean Ct values and coefficients of variation (CV) were calculated.

### 2.9. Clinical Application

A total of 151 clinical samples (including organisms, nasal swabs, feces, blood, etc.) were collected. were collected from various regions in Henan Province. The organism collected samples from some pigs that were experiencing reproductive disorders and respiratory symptoms. The feces and nasal swabs were collected from healthy pigs instead. Total nucleic acids were extracted from all samples using an automated nucleic acid extraction system. These nucleic acid samples were tested in parallel using both the quadruplex real‐time PCR assay developed in this study and standard methods (GETV Reference Standard Method: NY/T 4303‐2023 Diagnostic techniques for animal GETV infection; PRRSV Reference Standard Method: GB/T 18090‐2023 Diagnostic techniques for porcine reproductive and respiratory syndrome; PCV2 Reference Standard Method: GB/T 35901‐2018 Real‐time PCR method for detection of porcine circovirus type 2; JEV Reference Standard Method: SN/T 2472‐2010 Protocol of quarantine technique for Japanese encephalitis) for the four target pathogens to validate the clinical application performance of the new assay. The standard method referred to is available at https://std.samr.gov.cn/.

## 3. Results and Analysis

### 3.1. Optimization Results

**Figure 1:**
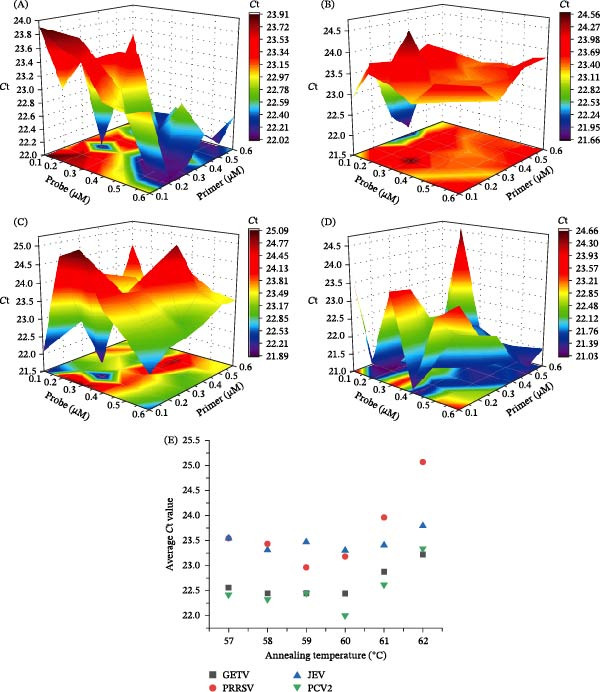
Optimization results for primer/probe concentrations and annealing temperature. (A) Results for GETV with primers at 0.3 µM and probe at 0.2 µM; (B) Results for PRRSV with primers at 0.4 µM and probe at 0.1 µM; (C) Results for JEV with primers at 0.2 µM and probe at 0.3 µM; (D) Results for PCV2 with primers at 0.2 µM and probe at 0.2 µM; (E) Determination of the optimal annealing temperature of 60°C for the quadruplex real‐time PCR assay.

To enhance the amplification efficiency of the quadruplex real‐time PCR assay, the annealing temperature, primer and probe concentrations, and cycle number were optimized using the matrix method and the single‐factor‐at‐a‐time approach. The results indicated that the optimal primer and probe concentrations for GETV, PRRSV, JEV, and PCV2 were 0.3 and 0.2 µM (Figure 1A), 0.4 and 0.1 µM (Figure 1B), 0.2 and 0.3 µM (Figure 1C), and 0.2 and 0.2 µM (Figure 1D), respectively, yielding the smallest Ct values under these conditions. Comprehensive consideration determined 60°C as the optimal annealing temperature (Figure 1E). There were 35 cycles with insufficient amplification, and for 45 and 50 cycles, the background values were too high, or there was nonspecific amplification. An amplification cycle number of 40 yielded the best performance and highest efficiency for the quadruplex real‐time PCR avoiding insufficient amplification or nonspecific amplification due to high background signals. The final reaction system is shown in Table 2. The optimal thermal cycling conditions were 52°C for 5 min, followed by 40 cycles of 95°C for 10 s, 95°C for 5 s, and 60°C for 25 s.

**Table 2:** Reaction system.

Component	Volume
2 × Fast One Step Probe RT‐qPCR Mix (Takara, China)	10 μL
GETV‐F/GETV‐R/GETV‐Probe	0.6 μL/0.6 μL/0.4 μL
PRRSV‐F/PRRSV‐R/PRRSV‐Probe	0.8 μL/0.8 μL/0.2 μL
JEV‐F/JEV‐R/JEV‐Probe	0.4 μL/0.4 μL/0.6 μL
PCV2‐F/ PCV2‐R/ PCV2‐Probe	0.4 μL/0.4 μL/0.4 μL
RNA	2.0 μL
RNase‐free Water	2.0 μL
Total	20 μL

### 3.2. Standard Curves

**Figure 2:**
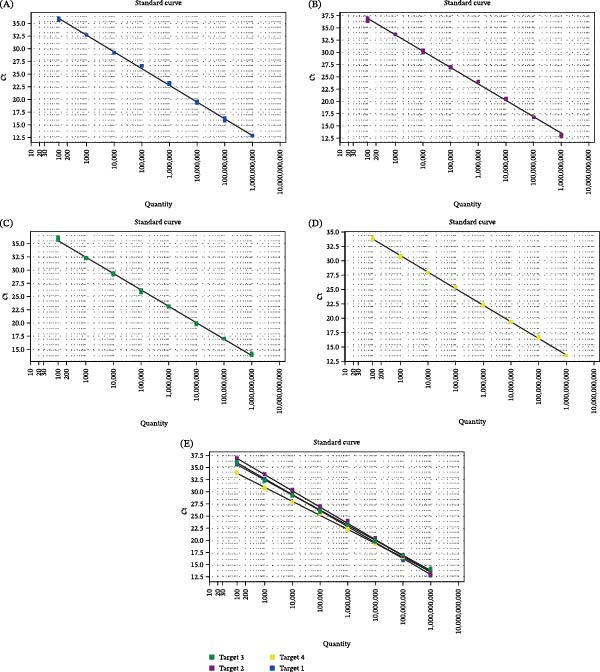
Standard curves of the quadruplex real‐time PCR assay. (A) GETV: linear equation *Y* = −3.3087 lg(*X*) + 42.102, *R* ^2^ = 0.9993, Eff = 101.286%; (B) JEV: linear equation *Y* = −3.4257 lg(*X*) + 43.103, *R* ^2^ = 0.9976, Eff = 98.626%; (C) PRRSV: linear equation *Y* = −3.225 lg(*X*) + 41.858, *R* ^2^ = 0.9972, Eff = 109.863%; (D) PCV2: linear equation *Y* = −2.918 lg(*X*) + 39.67, *R* ^2^ = 0.9995, Eff = 122.211%; and (E) Overlay of standard curves for GETV, JEV, PRRSV, and PCV2.

To evaluate the quantitative performance of the established quadruplex real‐time PCR assay, recombinant positive plasmids spanning a concentration gradient from 1 × 10^9^ to 1 × 10^2^ copies/μL were used as templates for amplification under optimized conditions. Standard curves were generated from QuantStudio 5 software. The results showed that the standard curves for GETV, PRRSV, JEV, and PCV2 exhibited good linearity, with *R* ^2^ values of 0.9993, 0.9972, 0.9976, and 0.9995, respectively. The amplification efficiencies were high, calculated at 101.286%, 109.863%, 98.626%, and 122.211%, respectively (Figure 2). Although the amplification efficiency of PCV2 was slightly above the ideal range (90%–110%), this was mainly due to the variability observed when detecting low‐concentration plasmid standards. Moreover, this study focused more on qualitative analysis rather than precise quantitative determination.

### 3.3. Limit of Detection (LOD)

**Figure 3:**
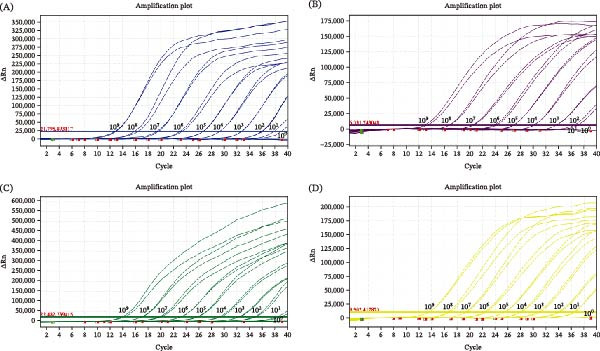
Detection results of positive plasmid standards with different gradient concentrations using the quadruple fluorescence quantitative PCR method. (A) GETV; (B) JEV; (C) PRRSV; and (D) PCV2.

**Figure 4:**
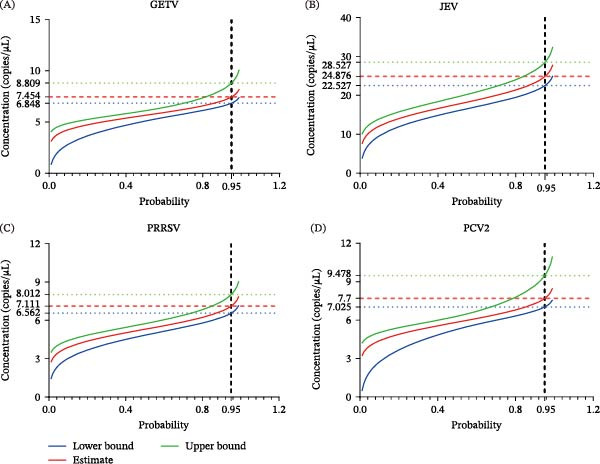
Probit regression analysis for the determination of the limit of detection (LOD) for the four pathogens: (A) LOD for GETV: 7.454 copies (95% confidence interval: 6.848–8.809); (B) LOD for JEV: 24.876 copies (95% confidence interval: 22.527–28.527); (C) LOD for PRRSV: 7.111 copies (95% confidence interval: 6.562–8.012); (D) LOD for PCV2: 7.7 copies (95% confidence interval: 7.025–9.478).

To evaluate the sensitivity of the assay, a two‐step procedure was employed to determine the LOD. First, to establish the approximate detection range, the recombinant plasmid standards for the four pathogens were subjected to tenfold serial dilution from 1 × 10^9^ copies/µL down to 1 × 10^0^ copy/µL and tested under optimized conditions. As shown in Figure 3, amplification curves and Ct values were observed for GETV, PRRSV, and PCV2 at 10 copies and for JEV at 100 copies. Subsequently, to precisely determine the LOD at a 95% confidence level, more refined twofold serial dilutions were prepared and analyzed using Probit regression. Specifically, concentrations of 50, 25, 12.5, 6.25, 3.125, and 1.5625 copies per reaction were tested for GETV, PRRSV, and PCV2, while concentrations of 200, 100, 50, 25, 12.5, and 6.25 copies per reaction were tested for JEV. Each concentration was assessed in 40 replicates. Table 3 presents the detection rates and average Ct values for each concentration. Probit regression analysis performed using SPSS software yielded LODs at a 95% confidence level of 7.454 copies for GETV, 24.876 copies for JEV, 7.111 copies for PRRSV, and 7.7 copies for PCV2 (Figure 4). When the Ct values of the FAM and PRRSV fluorescence channels are less than 39 and there is an amplification curve, it is determined that the GETV and PRRSV nucleic acids are positive. When the Ct value of the Cy5 fluorescence channel is less than 38 and there is an amplification curve, it is determined that the JEV nucleic acids are positive. When the Ct value of the JED fluorescence channel is less than 37 and there is an amplification curve, it is determined that the PCV2 nucleic acids are positive. When the Ct value is greater than the critical value and there is no amplification curve, it is determined to be negative for nucleic acids.

**Table 3:** Detection rates and mean Ct values of recombinant plasmid standards at different concentrations for the four pathogens.

Recombinant plasmids	Concentrations (copies/μL)	Samples	The quadruplex fluorescent quantitative PCR method	
			Ct (average)	Detection rate (%)
GETV	50	40	36.22	100
	25	40	37.19	100
	12.5	40	37.95	100
	6.25	40	38.76	87.5
	3.125	40	Undetermined^∗^	0
	1.5625	40	Undetermined	0
JEV	200	40	34.28	100
	100	40	36.07	100
	50	40	36.88	100
	25	40	38.23	95
	12.5	40	38.99	12.5
	6.25	40	Undetermined	0
PRRSV	50	40	36.44	100
	25	40	37.18	100
	12.5	40	38.32	100
	6.25	40	39.01	82.5
	3.125	40	39.55	5
	1.5625	40	Undetermined	0
PCV2	50	40	34.31	100
	25	40	35.66	100
	12.5	40	36.56	100
	6.25	40	37.44	77.5
	3.125	40	Undetermined	0
	1.5625	40	Undetermined	0

^∗^No Ct value.

### 3.4. Specificity Test Results

**Figure 5:**
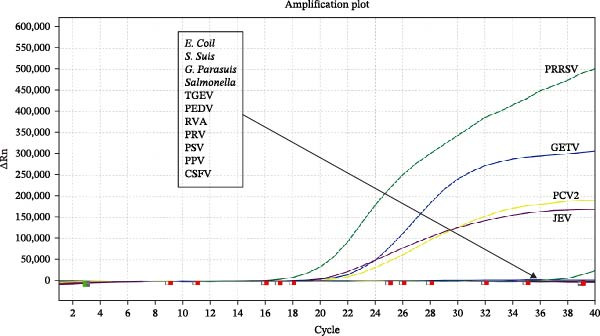
Specificity detection results of the multiplex quantitative PCR method.

To validate the specificity of the assay, nucleic acids from *S. suis*, *G. parasuis*, TGEV, PEDV, RVA, *E. coli*, *Salmonella*, PRV, PSV, PPV, and CSFV were amplified using the optimized quadruplex real‐time PCR method. GETV, PRRSV, JEV, and PCV2 served as positive controls, and nuclease‐free water served as the negative control. The results demonstrated that the established quadruplex real‐time PCR assay produced amplification curves only for the nucleic acids of the target pathogens and showed no cross‐reactivity with the other pathogens tested, indicating the high specificity of the method (Figure 5).

### 3.5. Repeatability Test Results

To evaluate the stability of the assay, intra‐assay and inter‐assay repeatability tests were performed using the established quadruplex real‐time PCR method. Recombinant plasmid standards (pMD18T‐GETV, pMD18T‐PRRSV, pMD18T‐JEV, and pMD18T‐PCV2) at three concentration gradients (1 × 10^7^, 1 × 10^5^, and 1 × 10^3^ copies/μL) were tested. Statistical analysis showed that the intra‐assay CV ranged from 0.13% to 1.09%, and the inter‐assay CV ranged from 0.17% to 1.39% (Table 4), indicating good repeatability of the method.

**Table 4:** Mean and coefficient of variation of Ct values of recombinant positive plasmid standards with different concentration gradients.

Standard plasmid	Concentration of template (copies/μL)	Intracoefficient of variation		Intercoefficient of variation	
		*X* ± SD	CV (%)	*X* ± SD	CV (%)
pMD18T‐GETV	10^7^	18.594 ± 0.025	0.13	18.921 ± 0.033	0.17
	10^5^	25.462 ± 0.104	0.41	25.422 ± 0.085	0.33
	10^3^	32.325 ± 0.141	0.44	32.621 ± 0.204	0.63
pMD18T‐PRRSV	10^7^	19.652 ± 0.200	1.02	19.365 ± 0.098	0.51
	10^5^	25.958 ± 0.196	0.76	26.013 ± 0.199	0.77
	10^3^	32.655 ± 0.294	0.90	32.551 ± 0.453	1.39
pMD18T‐JEV	10^7^	19.319 ± 0.132	0.68	19.225 ± 0.094	0.49
	10^5^	25.441 ± 0.079	0.31	25.364 ± 0.061	0.24
	10^3^	31.019 ± 0.112	0.36	31.212 ± 0.108	0.35
pMD18T‐PCV2	10^7^	19.553 ± 0.213	1.09	20.001 ± 0.120	0.60
	10^5^	25.046 ± 0.094	0.38	24.998 ± 0.108	0.43
	10^3^	30.331 ± 0.138	0.45	30.326 ± 0.237	0.78

### 3.6. Clinical Sample Detection Results

**Figure 6:**
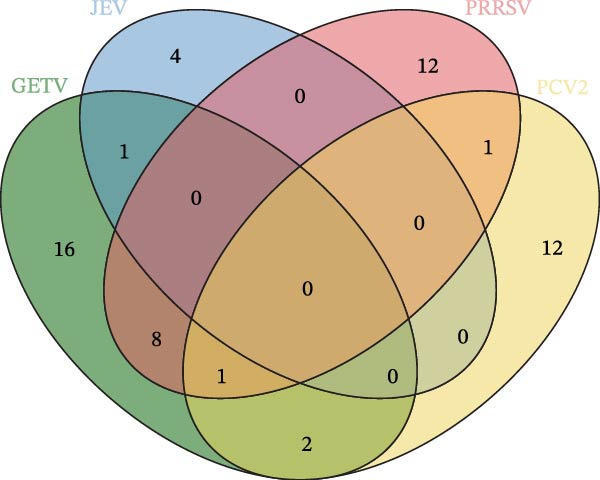
Results of single infection and mixed infection in positive clinical samples.

To validate the accuracy of the assay in practical applications, 151 clinical samples (including blood, nasal swabs, and feces) collected from various regions in Henan Province were tested using the established quadruplex real‐time PCR method. The results showed positive rates of 18.54% (28/151), 14.57% (22/151), 3.31% (5/151), and 10.60% (16/151) for GETV, PRRSV, JEV, and PCV2, respectively. The rates of mixed infections were as follows: 0.66% (1/151) for GETV and JEV coinfection, 5.3% (8/151) for GETV and PRRSV coinfection, 1.32% (2/151) for GETV and PCV2 coinfection, 0.66% (1/151) for PRRSV and PCV2 coinfection, and 0.66% (1/151) for GETV, PRRSV, and PCV2 triple infection (Figure 6). The results demonstrated 100% concordance when compared with established standard methods, confirming the high accuracy of the developed assay.

## 4. Discussion

GETV, PRRSV, JEV, and PCV2 are all significant pathogens that pose serious threats to the global swine industry. GETV, an emerging mosquito‐borne zoonotic virus, can cause fever, reproductive failure, and neurological signs in pigs [4]. PRRSV is the causative agent of Porcine Reproductive and Respiratory Syndrome (“blue ear” disease), leading to severe immunosuppression and respiratory disorders [7]. JEV, also a mosquito‐borne virus, not only causes reproductive failure in pigs but also poses a significant threat to public health [13]. PCV2 is a key pathogen responsible for postweaning multisystemic wasting syndrome (PMWS) and often acts synergistically with other pathogens [9]. The clinical manifestations of these pathogens are relatively similar, and mixed or secondary infections are extremely common. For instance, coinfection with PRRSV and PCV2 can exacerbate disease severity [11]. Currently, the trend of mixed infections involving GETV and other viruses in swine populations is also rising, as exemplified by the isolation of GETV from a PRRSV live vaccine by Gao et al. [14]. Therefore, establishing a detection method capable of rapidly and accurately identifying and monitoring these four pathogens is crucial for formulating prevention and control strategies and assessing public health risks.

In recent years, the isothermal amplification detection technology has demonstrated significant potential for on‐site testing due to its advantages of speed and convenience. Numerous reports have described its application for GETV, PRRSV, JEV, and PCV2. For example, an RT‐RPA‐CRISPR/Cas12a‐LFD detection method targeting the E2 gene of GETV can deliver results within 50 min with a LOD as low as 10 copies/μL [20]. Another study reported an RT‐LAMP method targeting the GETV E1 gene, exhibiting a sensitivity 10–100 times greater than that of conventional RT‐PCR [21]. For PRRSV detection, an RF‐RT‐RAA method completes the reaction in just 20 min at 42°C [22]. Furthermore, a combination of RT‐RPA and LFD for detecting PRRSV type 2 achieves a reaction time of 30 min at 42°C, with a sensitivity (5.6 × 10^−1^ TCID_50_/reaction) superior to that of conventional RT‐PCR [23]. Regarding PCV2 detection, combining CRISPR‐Cas12a/13a technology with ERA, RPA, or LAMP can achieve single‐copy level sensitivity [10, 24]. For instance, an RPA‐LFD method developed based on the ORF2 gene of PCV2 completes detection within 20 min at 37°C with a sensitivity of 10^2^ copies/reaction [24]. For JEV detection, a reported RPA method combined with CRISPR/EsCas13d can detect as few as 2 copies of viral nucleic acids [25]. Additionally, a developed RT‐RPA method combined with CRISPR‐Cas12a demonstrated limits of detection ranging from 10 to 10^2^ copies for different JEV genotypes [26]. Although these technologies are convenient, they are relatively costly and are limited to detecting only one pathogen per reaction. For intensive, large‐scale farms with high sample volumes and the need to screen for multiple pathogens, they struggle to meet the demands for efficient testing.

Real‐time quantitative PCR technology, with its maturity, strong specificity, high sensitivity, good reproducibility, and high‐throughput capability, remains one of the “gold standard” techniques for pathogen detection. Therefore, this study successfully established a quadruplex real‐time PCR method capable of simultaneously detecting GETV, PRRSV, JEV, and PCV2. We downloaded and aligned the genomic sequences of prevalent strains of GETV, PRRSV, JEV, and PCV2 from the NCBI database. Primers and probes were designed targeting highly conserved and specific regions and were labeled and distinguished using four fluorescent channels: FAM, ROX, Cy5, and JED. Through systematic optimization, an efficient amplification system and conditions were obtained. This method exhibits strong specificity, showing no cross‐reactivity with common swine pathogens such as PRV, CSFV, and *Streptococcus suis*. It demonstrates good stability, with both intra‐assay and inter‐assay CV below 1.4%. Furthermore, probit regression analysis determined the limits of detection for GETV, PRRSV, JEV, and PCV2 to be 7.454 copies, 24.876 copies, 7.111 copies, and 7.7 copies, respectively. The sensitivity is comparable to or higher than that of the reported single‐pathogen detection methods. For example, the SYBR Green I real‐time PCR method for GETV established by Xia et al. [27] had a LOD of 6.66 × 10^1^ copies/μL. The TaqMan real‐time PCR detection method developed by Lin et al. [28], based on the conserved region of the GETV E1 gene, showed strong specificity and good repeatability, with a detection limit of 5.94 copies/μL, which is 10 times more sensitive than conventional PCR. Current real‐time PCR methods for PRRSV are primarily used for typing. For instance, the duplex RT‐qPCR method developed by Tian et al. [7], based on the ORF6 gene of PRRSV‐1 and PRRSV‐2, can simultaneously differentiate between PRRSV‐1 and PRRSV‐2, with detection limits of 8.42 and 7.84 copies/reaction for the two genotypes, respectively. The quadruple RT‐qPCR method constructed by Ye et al. [29] for the Chinese epidemic situation, based on the NSP2 gene deletion characteristics, can simultaneously detect PRRSV‐2 and differentiate between three major lineages: the NADC30 strain, the highly pathogenic strain, and the NADC34 strain, with a detection limit of 3 copies/μL. For the detection of JEV, Freddi et al. [13] systematically evaluated three RT‐qPCR methods for swine wastewater surveillance and found that the ACDP JEV G4 assay performed optimally in terms of sensitivity (detection limit 2.20–5.70 copies/reaction) and field sample detection rate (23/30), providing the best choice for wastewater surveillance in areas with genotype 4 prevalence [13]. Furthermore, Liu et al. [12] successfully developed a triple RT‐qPCR method capable of simultaneously detecting JEV, Murray Valley encephalitis virus (MVEV), and West Nile virus (WNV), demonstrating a concordance rate of 93.9% to 100% with singleplex RT‐qPCR results. In the multiplex real‐time PCR assays established by Yin et al. [15] and Quan et al. [30], the limits of detection for PCV2 were both 10 copies/μL. Chen et al. [31] developed a TaqMan‐based triple real‐time PCR method that can simultaneously differentiate PCV2, PCV3, and PCV4. This method exhibited good specificity and reproducibility, with limits of detection of 53.3, 12.0, and 13.8 copies/μL for PCV2, PCV3, and PCV4, respectively [31]. In comparison, the sensitivity of the quadruplex real‐time PCR method established in this study for GETV, PRRSV, JEV, and PCV2 is equivalent to or higher than that reported for singleplex, duplex, and other multiplex qPCR assays.

In recent years, the prevalence of GETV, PRRSV, JEV, and PCV2 has shown a concerning trend. Outbreaks caused by the GETV GIII variant have been concentrated in regions such as Henan and Guangdong, China, with indications of increasing virulence and sustained mosquito‐borne transmission cycles [2, 32, 33]. PRRSV exhibits a complex cocirculation and rapid evolution of multiple lineages (e.g., the dominant lineage 1.8 and others), and coinfection with PCV2 is known to exacerbate disease severity [11, 34]. PCV2 itself maintains a high prevalence and is frequently involved in coinfections with pathogens like PCV3, PCV4, or PEDV [31, 35]. JEV exhibits endemic circulation with seasonal patterns in swine herds, and its virulence varies among different genotypes [36, 37]. Testing of 151 clinical samples collected from swine farms in Henan Province using the quadruplex real‐time PCR method established in this study revealed the highest detection rate for GETV (18.54%). Mixed infections involving GETV and other viruses were also detected, indicating complex patterns of multipathogen coinfection in clinical settings that warrant increased attention. However, the limited sample size in this study may not fully represent the prevalence of these four pathogens across swine farms. In the future, larger‐scale sample collection and continuous monitoring of these pathogens will be necessary to inform the development of targeted prevention and control strategies based on the epidemiological trends.

## 5. Conclusion

In this study, we successfully established a quadruplex real‐time PCR assay with strong specificity, high sensitivity, and good stability for the simultaneous detection of four important pathogens in swine: GETV, PRRSV, JEV, and PCV2. This method overcomes the throughput limitations of most existing rapid detection technologies and offers the advantage of accurate quantification. It enables efficient screening and differentiation of the aforementioned pathogens, thereby providing a reliable, cost‐effective, and efficient technical solution to address the increasingly complex trend of multipathogen coinfections; facilitate precise clinical diagnosis; and support large‐scale epidemiological surveillance.

## Author Contributions

He Zhang and Changyou Xia conceived the study and reviewed and edited the manuscript. Haojie Wang, Lihong Xue, He Zhang, and Changyou Xia designed the methodology. Bowen Shan, Shuyan Wu, Tongqin An, Changqing Yu, He Zhang, and Changyou Xia performed the investigation, visualization, and supervision. Haojie Wang, Lihong Xue, He Zhang, and Changyou Xia wrote the original draft.

## Funding

The research was supported by grants from the Heilongjiang Province Natural Fund Joint Guidance Project (Grant LH2024C059), the National Natural Science Foundation of China‐Youth Science Fund (Grant 32500449), the National Key R&D Program of China (Grants 2023YFF0724604 and 2023YFF0724603), and the Prevention and Control of Emerging and Major Infectious Diseases‐National Science and Technology Major Project (Grant 2025ZD01900703).

## Disclosure

All authors read and approved the final article and provided the corresponding author with written permission to be named in the article.

## Ethics Statement

One hundred fifty‐one clinical samples (including blood, nasal swabs, and feces) were collected from various regions in Henan Province. It is imperative to underscore that no additional harm or intervention was imposed on the animals involved in this study. The Institutional Review Board of the Harbin Veterinary Research Institute determined that this study is exempt from the requirement for ethical review or approval.

## Conflicts of Interest

The authors declare no conflicts of interest.

## Data Availability

The data that support the findings of this study are available from the corresponding author upon reasonable request. The data that support the findings of this study are not publicly available due to restrictions concerning the source of the animal sera.
